# Inducing Herd Immunity against Seasonal Influenza in Long-Term Care Facilities through Employee Vaccination Coverage: A Transmission Dynamics Model

**DOI:** 10.1155/2015/178247

**Published:** 2015-05-25

**Authors:** Aaron M. Wendelboe, Carl Grafe, Micah McCumber, Michael P. Anderson

**Affiliations:** Department of Biostatistics and Epidemiology, College of Public Health, University of Oklahoma Health Sciences Center, 801 NE 13th Street, CHB 323, Oklahoma City, OK 73104, USA

## Abstract

*Introduction*. Vaccinating healthcare workers (HCWs) in long-term care facilities (LTCFs) may effectively induce herd immunity and protect residents against influenza-related morbidity and mortality. We used influenza surveillance data from all LTCFs in New Mexico to validate a transmission dynamics model developed to investigate herd immunity induction. *Material and Methods*. We adjusted a previously published transmission dynamics model and used surveillance data from an active system among 76 LTCFs in New Mexico during 2006-2007 for model validation. We used a deterministic compartmental model with a stochastic component for transmission between residents and HCWs in each facility in order to simulate the random variation expected in such populations. *Results*. When outbreaks were defined as a dichotomous variable, our model predicted that herd immunity could be induced. When defined as an attack rate, the model demonstrated a curvilinear trend, but insufficiently strong to induce herd immunity. The model was sensitive to changes in the contact parameter *β* but was robust to changes in the visitor contact probability. *Conclusions*. These results further elucidate previous studies' findings that herd immunity may not be induced by vaccinating HCWs in LTCFs; however, increased influenza vaccination coverage among HCWs reduces the probability of influenza infection among residents.

## 1. Introduction

The risk of influenza-related morbidity and mortality is typically the highest among the elderly, particularly among residents of long-term care facilities (LTCFs) [1]. Vaccination against influenza in LTCFs is a priority and the Centers for Disease Control and Prevention (CDC) recommend that health care workers (HCWs) in these facilities get vaccinated against influenza annually [2]. In the US, influenza vaccination coverage among residents ranges from 0% to 100% [3] and averages 80% or higher [4, 5]. Coverage in Europe has been reported to vary from 50 to 90% [6, 7].

There are many questions regarding the effectiveness of vaccinating HCW in preventing outbreaks of influenza among residents. Mathematical transmission dynamics models have a long history of being applied to address such questions. Early pioneers of these methods include Sir Ronald Ross (1857–1932) [8], Kermack and McKendrick [9, 10], and Anderson and May [11]. In addition to contributing to a better understanding of herd immunity, these methods have been applied to understand the geospatial spread of influenza [12], the spread of drug resistance (including within nursing homes) [13, 14], and influenza vaccine efficacy [15].

Vaccinating HCWs against influenza in an effort to protect residents of LTCFs is a classic scenario in which epidemiologists and other public health professionals are relying upon herd immunity to protect a vulnerable population. As such, studies using transmission dynamics models have been conducted to determine the coverage level needed to induce herd immunity in these facilities. In fact, one modeling study in Netherlands concluded that herd immunity could not be induced in LTCFs [16]. However, based on the results from our surveillance activities in LTCFs in New Mexico, we felt the incremental change in the odds of detecting an outbreak in a LTCF suggested herd immunity could be induced [3]. Thus, we aimed to reconstruct the van den Dool et al. model [16] and check it against our observed surveillance data. We also aimed to investigate how the definition of an outbreak affected the model results, particularly in the context of evaluating herd immunity in small populations, such as LTCFs. Finally, we aimed to investigate additional parameters to which the model may be sensitive.

## 2. Methods

### 2.1. Data Collection

The population studied and surveillance activities are described in detail elsewhere [3]. However, we will briefly describe our surveillance activities here. There were 76 LTCFs in New Mexico. Given that influenza illness among residents of LTCFs is a reportable illness, we required the director of nursing at each facility to report the previous month's number of influenza illnesses among both residents and HCWs during the 2006-2007 influenza season. Thus, data were collected and analyzed with resolution at the monthly level. (The same methods are applied to the 2007-2008 influenza season; however, the second season's data were not incorporated in the model.) Each LTCF which failed to report within the first seven days of the month was contacted by up to two telephone calls to collect the data.

Influenza illness was defined by influenza-like illness, positive rapid antigen test for influenza, or positive viral culture for influenza. The number of residents ranged across each facility from 5 to 345 and the number of HCWs ranged from 11 to 250. Influenza vaccination among both residents and HCWs also varied from 0% to 100%.

### 2.2. Mathematical Model

We created a mathematical model for influenza transmission in LTCFs based on a model by van den Dool et al. [16]. The parameter values are shown in Table 1. While we followed their model design as closely as possible, we used different values for parameters where surveillance data from New Mexico were available, including the number of residents and HCWs, the vaccine coverage rates for residents (vaccine coverage rates for HCWs in the model were artificially varied from 0 to 100% in 10% intervals, but the actual values from the surveillance data were used for validation), the number of days and facilities modeled, and the size of the general population [17].

Specifically, we used SEIR compartmental models to model transmission between residents and HCWs in each facility and in the broader community. The population begins in the susceptible (S) compartment, moves to the exposed (E) compartment, then moves to the infectious (I) compartment, and then moves to the recovered (R) compartment.

Contact sufficient for disease transmission could occur in three ways among residents and HCWs: between two residents, between a HCW and a resident, and between two HCWs. This transmission between contacts was modeled stochastically by random sampling from a Bernoulli distribution with the mean set equal to the transmission probability for a given encounter. For each pair of individuals, a probability for close or casual contact is assigned (*p*_1_ and *p*_2_) to reflect the probability of transmission. To account for the greater probability that transmission occurs if the contact is close, *p*_2_ is always larger than *p*_1_. The types of contact differed by shift. For example, there was no contact between two residents during the night shift. Contact with visitors from the community was also modeled. In contrast to the stochastic SEIR model in the LTCFs which models a small population, the SEIR model for the community at large was developed as a deterministic model to simulate contact in a large population. Because the community contact parameter *β* was not explicitly stated, we therefore set it to equal 46% to match the results of the van den Dool et al. model. New patients admitted to the LTCF from the community enter with the probability of infection equal to influenza prevalence in the community, and “off-shift” HCWs become infected after making contact with infected community members according to probability *β*. The following system of differential equations defines the movement between compartments in the community:

 
*ds*/*dt* = −*λs*;
 
*de*/*dt* = *λs*–*σe*;
 
*di*/*dt* = *σe*–*γi*;
 
*dr*/*dt* = *γi*;
 force of infection *λ* = *βi*.

### 2.3. Parameters

Unless otherwise noted, all of the fixed parameters and the model design, including the transitions between compartments and associated probabilities, were kept identical to those described in the van den Dool et al. paper and are described in detail in that paper's supporting information Text S1 [16]. We have included the needed details of both the van den Dool model and our model for the convenience of the reader. Table 1 shows the values for each parameter and indicates whether it was the same as or different from the van den Dool model. The time step, or shift, was 8 hours, the discharge mortality rate was 1/425 per day, the rate of becoming infectious after infection was 1/1.4 per day, the infection recovery rate was 1/1.4 per day, and the fraction of HCWs immune due to cross protection was 30%. For probabilities of contact between patients and HCW, estimates were made for both contact per shift and the probability that each encounter was close. The probability of casual contact between patients was 7% and the probability that each contact was close was 6%; for HCW and patient contact, the probability of contact was 52% and the probability that each contact was close was 69%; for between HCW contacts, the probability of contact was 91% and the probability that each contact was close was 32%. The ratio of close to casual transmission probability was set at 2. Vaccine efficacy against infection for patients was 25% and the vaccine efficacy against infection for HCWs was 73%.

For the uncertain parameters (i.e., vaccine efficacy among patients, vaccine efficacy among HCWs, transmission probability of casual contact (*p*_1_), and the average number of visitors), the median values from the van den Dool model were used and reported in Table 1. These were analyzed in the van den Dool et al. paper and were found to have little impact on variation in their results. However, we did conduct sensitivity analyses on certain parameters. Specifically, we varied the population contact parameter, *β*, from 36% to 48% (in 4% increments) and for the average number of visitors, *g*, we varied from 0 to 1 per resident per eligible shift (day or evening; 0 to 2 total per resident per day).

Data collected monthly from New Mexico LTCFs from November through April (180 days) were used to model influenza transmission within each of 63 facilities with the number of residents, the number of employees, and the resident vaccine coverage specific to each facility for each month included in the model. (Thirteen of 76 facilities were not included in the model because data for at least one of the four variables was missing for all six months.) The HCW vaccine coverage at each facility for each month was not included in the model but was used for model validation. Missing data for the remaining facilities were approximated using the average values for the respective variables over the six months.

Influenza outbreaks were defined as a binary variable and a continuous variable. The binary definition was in accordance with the recommendation from the Infectious Disease Society of America (IDSA) and was defined as a single case of influenza in a resident in a LTCF [18]. The continuous definition was defined as an attack rate. To investigate the induction of herd immunity, we examined outcomes from (1) the observed data and (2) the model and then (3) compared the results from the modeled data to the observed data. For the observed data, Pearson correlation coefficients and corresponding *p* values were calculated to estimate the association between the vaccination coverage among HCWs and the attack rate among residents. For the modeled data, the model was run in 10% increments of HCW vaccination coverage levels from 0% increasing to 100%. The model was run 1,000 times at each incremental level and the influenza attack rates among residents were averaged at each incremental coverage level. Additionally, a general linear mixed model with a quadratic term was included to check for evidence of a curvilinear trend. To check the model's performance against the observed data, a *χ* ^2^ goodness of fit test was conducted. (A statistically significant result would indicate evidence that the model results are different from the observed results.) The mathematical model and all analyses were conducted using SAS 9.1.3 (Cary, NC) and R [19].

## 3. Results and Discussion

**Figure 1:**
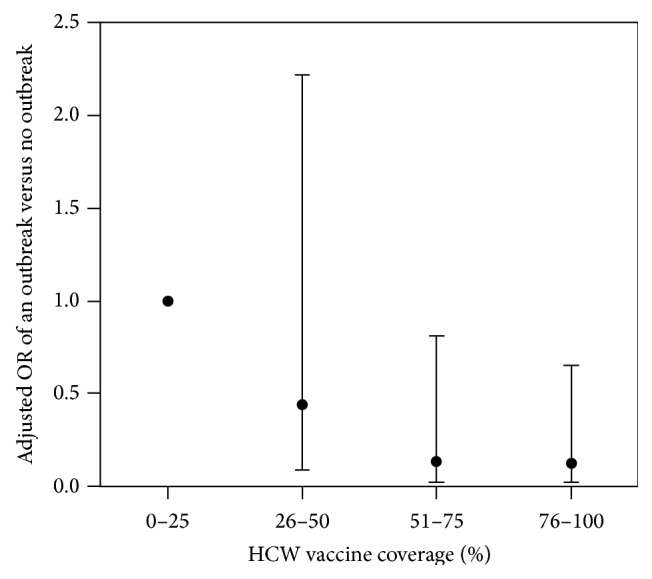
Adjusted odds ratios (and 95% confidence intervals) for detecting an influenza outbreak (dichotomized as outbreak = yes or no) and the proportion of health care worker influenza vaccination coverage categorized by quartiles in all long-term care facilities in New Mexico during the 2006-2007 and 2007-2008 influenza seasons.

**Figure 2:**
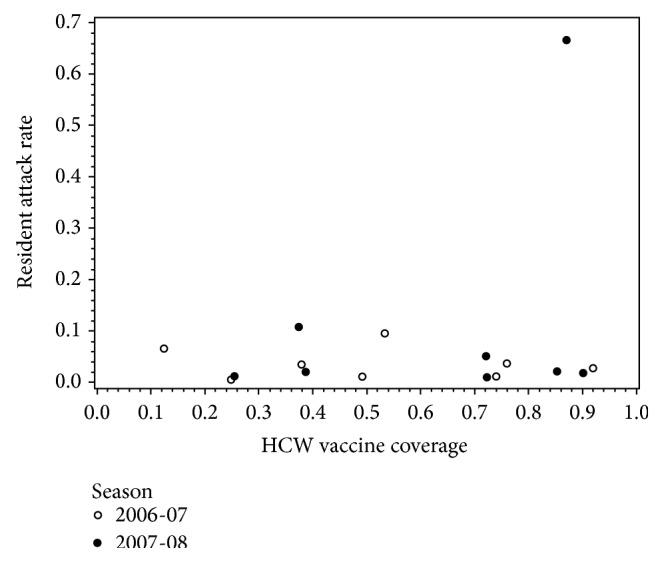
Association between influenza outbreaks defined on a continuous scale as the resident attack rate and the proportion of vaccination coverage among health care workers in all long-term care facilities in New Mexico during the 2006-2007 and 2007-2008 influenza seasons. There were nine outbreaks during 2006-2007; however, one outbreak was not plotted due to missing data for HCW vaccination coverage.

**Figure 3:**
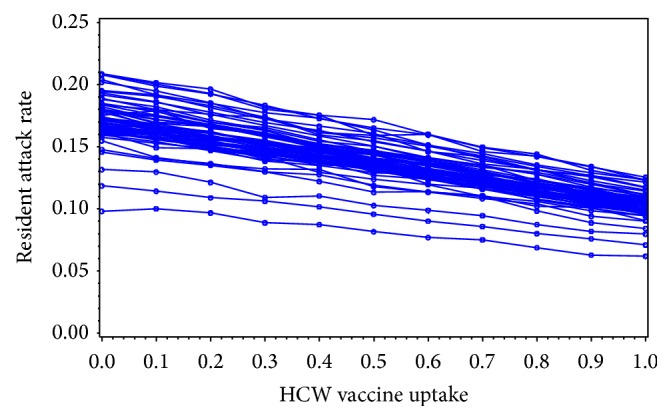
Results from general linear mixed model of resident influenza attack rates by HCW vaccination coverage for 63 LTCFs in New Mexico.

**Figure 4:**
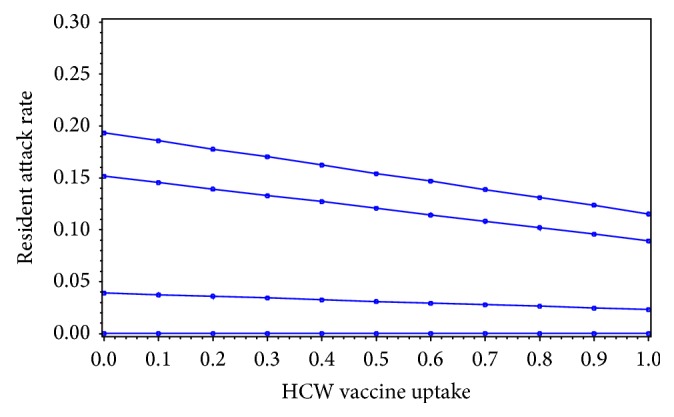
Results from general linear mixed model of average resident influenza attack rates by HCW vaccination coverage for 63 LTCFs in New Mexico with probabilities of contact in the community where transmission occurs of 0.36, 0.40, 0.44, and 0.48 (bottom to top).

**Figure 5:**
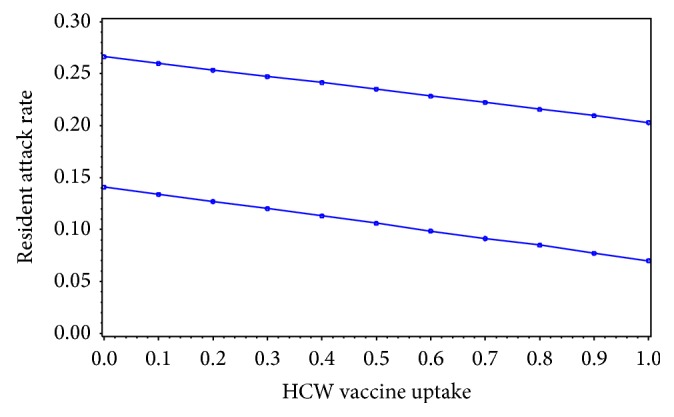
Results from a general linear mixed model of average resident influenza attack rates by HCW vaccination coverage for 63 LTCFs in New Mexico with average number of visitors from the community per resident per day of 0 (lower) and 2 (upper).

We found that the outcome definition of an influenza outbreak in a LTCF had a strong impact on whether herd immunity could be induced in LTCFs. Specifically, when defined as a dichotomous outcome (outbreak = yes or no), the odds of detecting an outbreak in logistic regression shows a decreasing curvilinear trend as HCW vaccination coverage increases (Figure 1). However, when influenza outbreaks were defined on a continuous scale as the resident attack rate, the trend was less striking. When examining the observed data, there was no clear correlation between increasing HCW vaccination coverage and decreasing influenza attack rates among residents (*r* = 0.25, *p* = 0.35) (Figure 2). The attack rates generated by the model ranged from 6.0% to 21.8% over the span of HCW vaccination coverage levels. We detected a curvilinear trend, with a quadratic term of −0.0018 (*p* = 0.027), suggesting that herd immunity could be induced. In addition, as vaccination uptake increased among health care workers, the magnitude of the influenza attack rate among residents decreased (Figures 3–5). However, as shown in Figure 3, the strength of the effect was too weak to actually achieve a herd immunity threshold lower than 100%. The result from our goodness of fit statistic was *χ* ^2^ = 85.7 (*p* = 0.025), providing evidence that the attack rates generated by the model were significantly different from the observed attack rates.

In sensitivity analyses, we found the model sensitive to the contact (*β*) parameter (i.e., the probability of transmission occurring given that the contact is infectious) of the community population. Specifically, small reductions (i.e., 2%–4%) reduced the size of the influenza attack rate in the LTCF. However, reducing *β* did not change the shape of the curvilinear trend or reduce it enough to induce the herd immunity threshold (Figure 4).

In an additional sensitivity analysis, we varied the average number of visitors per resident per day from 0 to 2 and found that it had little effect on the model's ability to induce herd immunity, though it did change the magnitude of the attack rate among residents (Figure 5).

Overall, we found that when outbreaks were defined as attack rates, a continuous measure, there was no evidence that herd immunity could be induced to protect residents of LTCFs by vaccinating HCWs, conclusions consistent with the prior study simulated in Netherlands [16]. In contrast, if the outcome is defined using a dichotomous measure and the odds of detecting an outbreak are used to estimate the association with HCW vaccination coverage levels, the possibility for herd immunity induction appears possible. We also found that increased influenza vaccination coverage among HCWs in LTCFs is instrumental in reducing the magnitude of attack rates of influenza among residents in LTCFs.

The findings from this study contribute to our understanding of the role of vaccinating HCWs in LTCFs in each of the following important ways: (1) we showed that the results of the model are sensitive to the definition of an outbreak of influenza (i.e., continuous versus dichotomous), (2) we were able to run a transmission dynamics model against observed surveillance data and thus help validate the previous model, and (3) we provided additional information on certain parameters not previously examined. Specifically, we demonstrated that even if we assumed that visitors did not have contact with residents sufficient to transmit influenza to residents, that herd immunity could not still be induced. However, a related, but slightly different, parameter, *β*, or probability of infectious contact between two individuals plays an important role in both the magnitude of the attack rate and the slope of the effect.

Our study is limited by certain factors. First, we incorporated only one season of surveillance data for influenza. However, influenza is a disease with such strong variability from season to season; our findings may not be representative of all influenza seasons. For example, the pathogenicity and virulence of the seasonal influenza strain vary each season, as does the effectiveness of the vaccine, such that it is possible that, during the influenza season under surveillance, the influenza vaccine was insufficiently effective to induce a herd immunity threshold. In addition, there is a relatively large amount of variation in the observed data compared to the modeled data (which was an average of 1,000 modeled seasons), making it difficult to interpret the goodness of fit statistic. Second is the small sample size of observed outbreaks. Even though we were able to conduct surveillance among all LTCFs in New Mexico, there were only nine influenza outbreaks during the 2006-2007 season. Third is the effect of missing data on the model. There were 13 facilities which never reported despite multiple efforts to solicit reports. Although it is likely that these facilities did not have an influenza outbreak during this surveillance period (because had an outbreak occurred, there would have been motivation for the facility to solicit help from the state health department to assist in infection control), it is impossible to be certain.

## 4. Conclusions

The measured benefit of vaccinating HCWs against influenza as a means of inducing herd immunity and thereby protecting residents of herd immunity depends on how influenza outbreaks are measured, as a binary event or as a continuous measure (attack rate). Regardless of the outcome measure, we observed that increasing influenza vaccination coverage among HCWs in LTCFs provides indirect protection to the residents, but using the continuous measure, it was insufficient to induce herd immunity. Thus, even in the absence of inducing herd immunity, following the recommendation that all HCWs should be vaccinated against influenza is associated with lower influenza attack rates among residents. In addition, reducing visitors' probability of transmitting influenza to residents is an important component of influenza disease control in LTCFs. More surveillance data in more locations is needed to further validate this transmission dynamics model. In addition, future model building should include sensitivity analyses which identify the key parameter(s) capable of inducing herd immunity in small populations such as LTCFs.

## Figures and Tables

**Table 1:** The parameter values for each variable in the SEIR compartmental model. Surveillance data refers to values obtained from conducting surveillance among each of the long-term care facilities in New Mexico during the 2006-2007 influenza season.

Parameter	Parameter value (range) Current study	Parameter value van den Dool et al. [16] study
Number of residents	Surveillance data (5–345)	30
Number of HCWs	Surveillance data (12–207)	30
Vaccination coverage among residents	Surveillance data (0%–99%)	0.75
Vaccination coverage among HCWs	Surveillance data (0–100%)	0-1
Number of days	120 (surveillance period)	80
Number of facilities	63	1
Size of general population	1,937,916 [17]	100,000
Contact (*β*)	46% (36%–48%)	Unknown
Average number of visitors	0.7 (0–2) per resident per day	0.7 (0.4–1) per resident per day
Time step	8 hours	8 hours
Discharge mortality rate	1/425 per day	1/425 per day
Infectious rate	1/1.4 per day	1/1.4 per day
Recovery rate	1/1.4 per day	1/1.4 per day
HCWs immune from cross protection	30%	30%
Probability of casual contact between patients	7%	7%
Probability of close contact between patients	6%	6%
Probability of casual contact between HCW and patient	52%	52%
Probability of close contact between HCW and patient	69%	69%
Probability of casual contact between HCWs	91%	91%
Probability of close contact between HCWs	32%	32%
Ratio of close to casual transmission probability	2	2
Vaccine efficacy among patients	25%	25%
Vaccine efficacy among HCWs	73%	73%

HCW: healthcare worker.
